# Susceptibility to DNA Damage as a Molecular Mechanism for Non-Syndromic Cleft Lip and Palate

**DOI:** 10.1371/journal.pone.0065677

**Published:** 2013-06-12

**Authors:** Gerson Shigeru Kobayashi, Lucas Alvizi, Daniele Yumi Sunaga, Philippa Francis-West, Anna Kuta, Bruno Vinícius Pimenta Almada, Simone Gomes Ferreira, Leonardo Carmo de Andrade-Lima, Daniela Franco Bueno, Cássio Eduardo Raposo-Amaral, Carlos Frederico Menck, Maria Rita Passos-Bueno

**Affiliations:** 1 Human Genome Research Center, Institute for Biosciences, University of São Paulo, São Paulo, Brazil; 2 Dental Institute, Department of Craniofacial Development and Stem Cell Biology, King’s College London, London, United Kingdom; 3 Institute of Biomedical Sciences, University of São Paulo, São Paulo, Brazil; 4 SOBRAPAR Institute, Campinas, São Paulo, Brazil; University of Miami, United States of America

## Abstract

Non-syndromic cleft lip/palate (NSCL/P) is a complex, frequent congenital malformation, determined by the interplay between genetic and environmental factors during embryonic development. Previous findings have appointed an aetiological overlap between NSCL/P and cancer, and alterations in similar biological pathways may underpin both conditions. Here, using a combination of transcriptomic profiling and functional approaches, we report that NSCL/P dental pulp stem cells exhibit dysregulation of a co-expressed gene network mainly associated with DNA double-strand break repair and cell cycle control (p = 2.88×10^−2^–5.02×10^−9^). This network included important genes for these cellular processes, such as *BRCA1*, *RAD51*, and *MSH2*, which are predicted to be regulated by transcription factor E2F1. Functional assays support these findings, revealing that NSCL/P cells accumulate DNA double-strand breaks upon exposure to H_2_O_2_. Furthermore, we show that *E2f1*, *Brca1* and *Rad51* are co-expressed in the developing embryonic orofacial primordia, and may act as a molecular hub playing a role in lip and palate morphogenesis. In conclusion, we show for the first time that cellular defences against DNA damage may take part in determining the susceptibility to NSCL/P. These results are in accordance with the hypothesis of aetiological overlap between this malformation and cancer, and suggest a new pathogenic mechanism for the disease.

## Introduction

Non-syndromic cleft lip with or without cleft palate (NSCL/P [OMIM %119530]) is one of the most common congenital defects. Its birth prevalence is variable, ranging from 3.4 to 22.9 per 10,000 births world-wide, depending upon factors such as ethnic background, geographical location, and socio-economic status [1].

The interplay between genetic and environmental factors during embryonic development is thought to be determinant in the aetiology of NSCL/P. Genome-wide association studies (GWAS) have enabled the consistent identification of several candidate *loci*. However, the low attributed risk for each variant fails to explain the estimated heritability for NSCL/P (as high as 85% in some populations [2]), and little is known about their functional role in the pathogenesis of the disease. The fact that non-coding genomic regions harbour many of these variants (which include an enhancer upstream of *IRF6*, and intronic and intergenic regions [3], [4], [5], [6] is indicative that they might play a transcriptional regulatory role in the embryo, in agreement with the idea that alterations in gene expression may be a relevant mechanism underlying susceptibility to complex diseases [7]. Therefore, if disease susceptibility is shaped by transcriptional anomalies, which in turn are driven by the individual’s genetic constitution, a feasible approach to investigate the aetiology of NSCL/P is the gene expression analysis of cells from affected individuals. This strategy not only allows the screening of candidate biological pathways contributing to the disease, but also enables the investigation of environmental agents and how they affect these pathways. Previous data derived from fibroblasts and mesenchymal stem cells support this rationale, as they revealed alterations in gene networks functionally relevant for orofacial development, such as collagen metabolism and extracellular matrix remodelling [8], [9], [10], [11].

NSCL/P is thought to arise from anomalies in cell migration, proliferation, transdifferentiation and apoptosis [12], [13], [14], all of which are known to be involved in cancer progression. The relationship between orofacial clefts and cancer is subject of debate; however, several studies have reported co-occurrence of orofacial clefts and a variety of cancer types. [15], [16], [17], [18]. Accordingly, alterations in genes that are known to play diverse roles during carcinogenesis, such as *CDH1*, *TP63*, *NBS* and *AXIN2*, have been related to both syndromic and non-syndromic cleft lip/palate [19], [20], [21], [22], [23], [24]. Moreover, both are common diseases with significant genetic heterogeneity; therefore, an aetiological overlap is more likely to occur when compared to other diseases. Given these observations, similar biological pathways may be underpinning susceptibility to both conditions.

Thus, our objective was to search for dysregulated gene networks associated with tumourigenesis, using NSCL/P and control dental pulp stem cell cultures. We chose this cell source because it comprises populations of mesodermal and neural crest-derived cells, and therefore it shares the same origin with the cells that populate the mesenchyme of the craniofacial structures involved in lip and palate morphogenesis [25], [26], [27]; in addition, we have previously demonstrated the applicability of using these cells in detecting gene networks important for NSCL/P aetiology [9]. We expect that the results will not only assist in elucidating the aetiology of NSCL/P, but also provide more information on the mechanisms through which it relates to cancer.

## Results

### Overview of the Differentially Expressed Genes (DEGs)

By comparing the microarray expression data from NSCL/P (n = 7) and control (n = 6) dental pulp stem cell cultures, we obtained 126 and 211 DEGs using the SAM (Significance Analysis of Microarrays) and Rank Products algorithms, respectively. Combining these two gene sets, we observed an overlap of 109 genes from both methodologies, with a final number of 228 DEGs (72 up-regulated, 156 down-regulated; Table S1), which were submitted to the subsequent analyses.

### A Transcriptional Network Associated with Response to DNA Damage and Cell Cycle Control is Dysregulated in NSCL/P Cells

**Figure 1:**
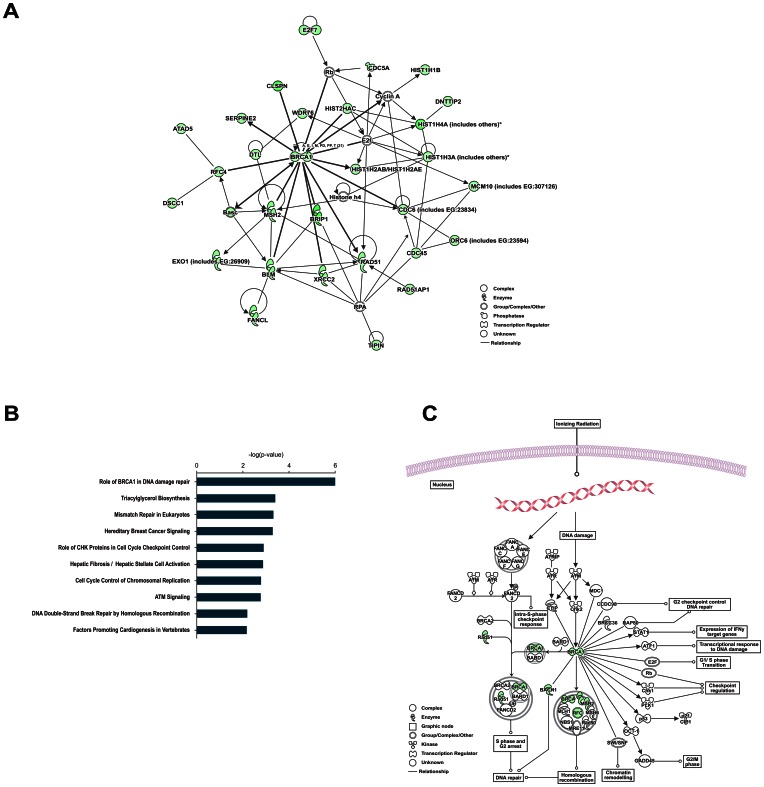
NSCL/P cells exhibit a gene expression profile associated with dysregulation of DNA repair and cell cycle control. (A) IPA network. DEGs were used to assemble a functional interaction map. Lines with arrowheads represent that one molecule acts on another, while regular lines represent protein interactions. Down-regulated genes are shown as green nodes, whereas genes without differential expression are shown as blank nodes. (B) Top 10 Canonical Pathways significantly enriched among the 228 DEGs (Fisher’s Exact Test, p-value <0.01). (C) ‘Role of BRCA1 in DNA Damage Response’ Canonical Pathway.

To functionally characterise the DEGs and to determine possible interactions between them, an IPA analysis (Interactive pathways analysis of complex ‘omics data; Ingenuity Systems) was performed. The highest-scoring network assembled by IPA (score = 61) harboured DEGs associated with the following functions: Cell Cycle; DNA Replication, Recombination, and Repair; and Cellular Compromise (p = 2.88×10^−2^–5.02×10^−9^; Table S2), in which the gene *BRCA1* occupied a central node, functionally connected to a variety of other molecules associated with DNA repair and cell cycle regulation (e.g. *MSH2*, *BLM*, *RAD51*, *CDC6, CLSPN*; Fig. 1A). Moreover, the top canonical pathway enriched in our gene set was ‘Role of BRCA1 in DNA Damage Response’ (p-value = 3.92×10^−8^; Fig. 1B and 1C). Taken together, the results indicated that NSCL/P cells feature transcriptional dysregulation of genes involved in cell cycle control and DNA damage repair mediated by BRCA1. This motivated us to further investigate this molecule and its relationship with the remaining DEGs.

**Figure 2:**
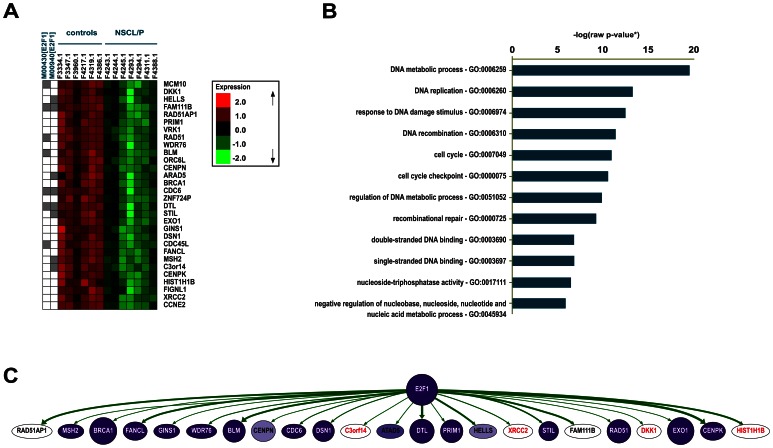
NSCL/P patterns of co-expression are associated with DNA repair, and suggest E2F1 as a regulator. (A) Supervised similarity cluster based on *BRCA1* expression (avg. homogeneity = 0.974). Transcription factor binding sites significantly over-represented in the cluster are marked in grey, for each motif identified (Bonferroni-adjusted p-value <0.05). (B) GO attributes enriched in the similarity cluster and their respective representation among the 30 clustered genes, expressed in percentages ([*] Bootstrap-adjusted p-values = 0.001, raw p-values were used in the chart). (C) Analysis of transcription factor-gene interactions. ChIP-chip data from FANTOM4 were used to validate the interaction between E2F1 and 23 out of the 30 genes of the similarity cluster. The thickness of the arrows indicates how often the interaction has been experimentally detected; node colours represent the level of expression in the cell lines used to assemble the database, from low (light) to high (dark).

Assuming that co-expressed genes may partake in the same biological process, we performed a similarity-based clustering analysis, based on *BRCA1* transcript levels. We obtained a highly homogeneous cluster harbouring 30 genes of similar expression patterns across samples (average homogeneity = 0.974, Fig. 2A), including several genes pertaining to the IPA interaction network and BRCA1-mediated DNA repair canonical pathway, such as *MSH2*, *RAD51*, and *BLM*. In accordance with the observations derived from IPA, this cluster exhibited Gene Ontology enrichment for attributes related to the DNA repair machinery and regulation of cell cycle (p = 0.001; Fig. 2B). Subsequent transcription factor binding site enrichment analysis revealed E2F1 as a putative regulator for this co-expression cluster (p<0.05; Fig. 2B). To corroborate this result, we used ChIP-chip information available on the FANTOM4 (Functional ANnoTation of the Mammalian genome) database, demonstrating that E2F1 is experimentally proven to interact with 23 out of all 30 clustered genes (Fig. 2C).

### Validation of the Microarray Assays using Quantitative Real Time PCR (qRT-PCR)

We carried out the qRT-PCR validation using RNA extracted from independent cell cultures from the same individuals submitted to the microarray assays. We applied this strategy as an attempt to avoid biased interpretation of the transcriptomic data, as the expression of many of the genes detected is cell-cycle dependent and therefore may be subject to fluctuations in asynchronous cultures.

Twenty-four DEGs, including 10 genes present in the *BRCA1* similarity cluster, were submitted to validation by qRT-PCR. We observed that one NSCL/P sample (F4243.1) exhibited a discordant expression pattern for 15 out of the 24 genes as compared to the rest of the NSCL/P group, with expression values distant at least 3 standard deviations from the mean (data not shown). Based on this observation and the fact that RNA aliquots did not correspond to the original samples hybridised on the microarray chips, we classified this sample as an outlier and excluded it from the subsequent analyses. Differences in expression values between NSCL/P and control samples were tested for statistical significance, which revealed that mRNA levels for 17 out of the 24 DEGs selected for validation were significantly different between groups (p<0.05). Moreover, we confirmed the differential expression of all genes that were submitted to validation and that also pertained to the *BRCA1* similarity cluster (Table S3).

### NSCL/P Cells Accumulate Double-strand Breaks (DSBs) upon Exposure to H_2_O_2_

**Figure 3:**
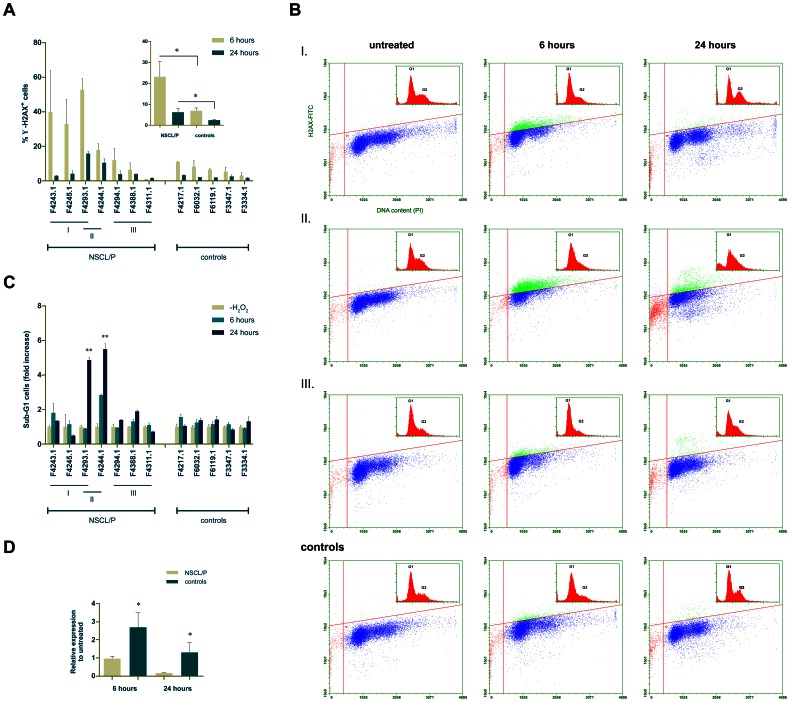
NSCL/P cells exhibit a heterogeneous response to H_2_O_2_, with distinct patterns of DSB accumulation. (A) Smaller graph - Comparison of the fraction of γ-H2AX^+^cells between NSCL/P and control cells, at 6 and 24 hours of treatment with H_2_O_2_. Large graph – Individual quantitation of γ-H2AX^+^ cells, revealing the subgroups (I–III) within the NSCL/P samples. (*) p<0.05. (B) Representative γ-H2AX and PI profiles depicting DSB and cell cycle distribution, for each NSCL/P subgroup and controls. (C) Quantitation of sub-G1 cells, revealing a significant increment in NSCL/P subgroup II, at 24 hours of treatment, as compared to the other subgroups and the controls. (**) F = 6.04; p<0.005. (D) Relative expression of *CDC45L* following treatment with H_2_O_2_, revealing a significant decrease in NSCL/P samples at both time points, by comparison to controls. (*) p<0.05.

In the presence of DNA double-strand breaks (DSBs), the BRCA1 pathway is responsible for cell cycle checkpoint regulation, DNA damage sensor signalling, and participates in DNA repair through homologous recombination [28]. Moreover, oxidative stress plays a major role in carcinogenesis and in teratogen-induced congenital malformations through DSBs induced by reactive oxygen species [29]. Based on the microarray results, we asked if the transcriptional dysregulation of the DSB response system would result in observable cellular phenotypes in NSCL/P cells upon oxidatively generated DNA damage. By flow cytometry quantitation of γ-H2AX (phospho-histone H2AX), we assessed DSB formation in cell cultures exposed to H_2_O_2_, using 7 NSCL/P and 5 control samples. We observed that a significantly greater proportion of NSCL/P cells were positively stained for γ-H2AX (γ-H2AX^+^ cells; quantitated in relation to untreated cells for each individual) after 6 and 24 hours of H_2_O_2_ treatment, when compared to controls (p<0.05; Fig. 3A, smaller graph). In fact, NSCL/P samples exhibited a heterogeneous response to H_2_O_2_ treatment, which could be divided into three subgroups: some individuals exhibited a very high percentage of γ-H2AX^+^cells after 6 hours (subgroup I - F4243.1, F4245.1, F4293.1); a second subgroup maintained a higher frequency of γ-H2AX^+^ for longer (24 hours; subgroup II - F4244.1 and F4293.1); and a third subgroup exhibited a lower frequency of γ-H2AX^+^ cells, similar to the control cells (subgroup III - F4294.1, F4311.1 and F4388.1), as shown in figure 3A and 3B. For subgroup II, we observed an accumulation of γ-H2AX^+^ cells in G1 and early S that coincided with the still elevated γ-H2AX staining at 24 hours. This subgroup also exhibited a significant increment of sub-G1 cells in this time point, compared to controls and the other subgroups (p<0.005; Fig. 3C). Therefore, subgroup II had problems to resume the cycle even at 24 hours, and these cellular responses to H_2_O_2_ treatment indicate that at least part of the NSCL/P cells (subgroup II) presents a defective repair of DSBs, and this leads to increased cell death.

Next, using an expanded sample of NSCL/P (n = 11) and control (n = 10) cells, we performed qRT-PCR experiments to assess if exposure to H_2_O_2_ affects the transcriptional behaviour of DEGs related to cell cycle control and DNA repair that were co-expressed with *BRCA1*, and also the putative upstream regulator *E2F1*. We found that after 6 hours of treatment *E2F1*, *CDC6*, *BRCA1*, *BRIP1*, *RAD51*, *RAD51AP1*and *BLM* did not exhibit significant differences in fold-expression compared to untreated samples, for both NSCL/P and control groups. These genes were transcriptionally repressed at 24 hours, possibly as a cellular response to the treatment. On the other hand, *CDC45L*, which is essential for new DNA synthesis, was more expressed in control cells after 6 hours and 24 hours when compared to NSCL/P cells (p<0.05, Fig. 3D).

In light of these findings, we proposed that NSCL/P cells also feature abnormalities in the response to oxidatively generated DNA damage. Therefore, we went back to the transcriptomic data and searched for DEGs related to oxidative stress and subsequent steps of oxidatively generated DNA damage repair. We found differential expression of several molecules directly or indirectly related to these processes, including genes involved in oxidative stress (*GSTM2* [30], *NOX4* [31], *PTGS2* [32], *SMAD3* [33], *BRCA1* [33]), stabilisation of the replication fork (*CLSPN* [34], *TIPIN* [34]), regulation of base-excision repair (*BRCA1* [35]), and new DNA synthesis during homologous recombination repair (*CDC45L* [36], *GINS1* [36], *MCM10* [37]; Table S4), in addition to those already found to be involved in homologous recombination (e.g. *RAD51, MSH2,* BLM; Table S1 and Fig. 1C).

### Murine Palatal Shelves Express DNA Damage Repair Genes

As the transcriptome analysis and functional assays were conducted in adult stem cells, we considered it crucial to verify if genes involved in DNA repair and cell cycle regulation are expressed in the embryonic structures important for lip and palate morphogenesis. Hence, using qRT-PCR, we first assessed expression of some of these genes, namely *Brca1*, *Brip1*, *Msh2*, *Rad51*, *Rad51ap1*, and *Blm*, in murine palatal shelves extracted at E11.5 (n = 2), E14.5 (n = 3), and E17.5 (n = 3). We observed a positive expression during palatal shelf growth, followed by a trend towards down-regulation of these genes after palate formation had been completed (E17.5), albeit with statistical significance only for *Rad51ap1* (p<0.05; Fig. S1), probably due to sample size.

**Figure 4:**
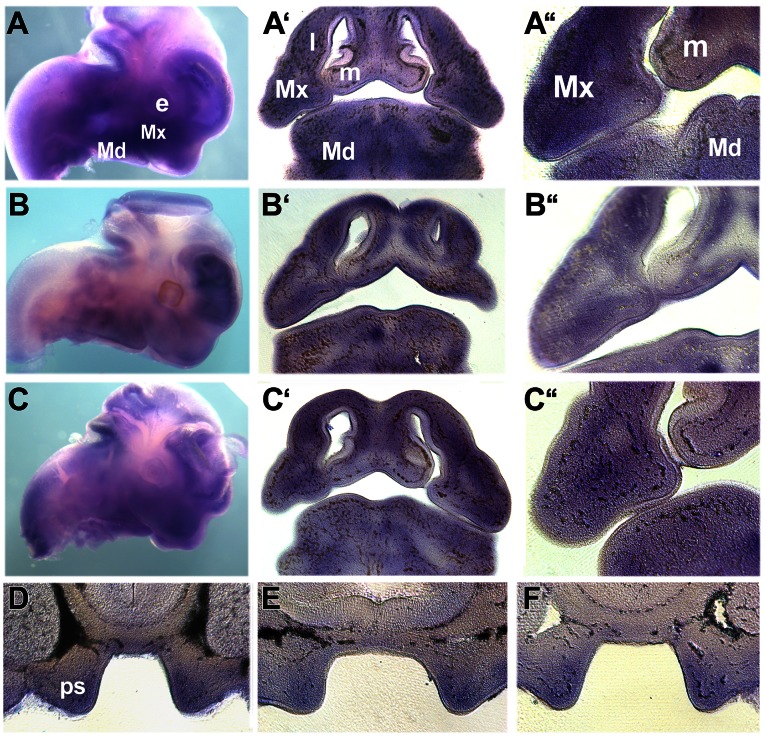
Expression of *Brca1*, *Rad51* and *E2f1* in the developing facial primordia. Whole-mount *in situ* hybridisation (A–F) showing the expression of *Brca1* (A, D), *Rad51* (B, E) and *E2F1* (C, F) in E11.5 (A–C) and E12.5 (D–F) embryos. Expression is indicated by the blue/purple staining. A–C are sagittal views of the developing head whilst A’–C’ are frontal sections through the embryos shown in A–C. A’’–C’’ show high power views through the developing maxillary primordia, lateral and medial nasal processes. E–F are frontal sections through the developing E12.5 palatal shelves. *e*, eye; *l*, lateral nasal process; *m*, medial nasal process; *Md*, mandibular primordia; *Mx*, maxillary primordia; *ps*, palatal shelves.

We also analysed *Brca1*, *Rad51*, and *E2f1* expression by whole-mount RNA *in situ* hybridisation of E10.5–E13.5 murine embryos. This revealed that *Brca1*, *Rad51* and *E2f1* transcripts are expressed in the mesenchyme of all the facial primordia (Fig. 4A-C and data not shown). Analysis of E11.5 embryos showed that all 3 genes are expressed in similar domains within the mesenchyme of the maxillary primordia, lateral and medial nasal processes which contribute to the development of the upper lip. Low levels of expression were also detected in the ectoderm at the point of fusion. At E12.5 and E13.5, *Brca1*, *Rad51* and *E2f1* transcripts were clearly co-expressed in the mesenchyme and in some regions of the ectoderm within the developing palatal shelves which arise in the maxillary primordia (Fig. 4E-F and data not shown). Negative sense hybridisation control showed no staining in the facial primordia (Figure S3).

## Discussion

Orofacial morphogenesis is dependent upon tightly regulated spatio-temporal patterns of cell migration, proliferation, transdifferentiation, and apoptosis [12], [13], [14]. Dysregulation of biological pathways orchestrating these processes is thus presumed to play a role in the pathogenesis of NSCL/P. Importantly, the identification of these pathways and how they interact with environmental agents will not only provide insight into the molecular basis of NSCL/P, but will also enable the development of more effective preventive strategies.

Using dental pulp stem cells from NSCL/P individuals, we identified a dysregulated transcriptional network mainly associated with response to DNA damage and cell cycle control. The functional interaction network assembled with the DEGs had a central node occupied by tumour suppressor *BRCA1* which, in combination with other key genes, plays a pivotal role in the cellular response to DNA damage and cell cycle control [38]. We confirmed that many genes found to be functionally connected to *BRCA1* in this network not only had a similar expression pattern, but also have well-established roles in the aforementioned cellular functions (e.g. *CDC6*, *CDC25A*, *MSH2*, *BLM, RAD51* [39], [40]). E2F1, the putative upstream regulator identified for this dysregulation block, is a transcription factor that acts in conjunction with its repressor pRB (encoded by *RB1*) and is responsible for up-regulating a variety of genes necessary for the transition from G1 to S in the cell cycle, being essential for cell cycle progression and DNA damage response [41], [42]. Also, it has been shown that E2F1 may play a role during murine palatogenesis [43]. Since *E2F1* and *RB1* were not differentially expressed in NSCL/P cells, we believe that disturbances at the protein level (i.e. affecting protein function but not necessarily the expression) could be responsible for altering the activity of these regulators, resulting in the expression patterns detected here.

The cellular functions attributed to the dysregulated gene network found here are strongly associated with tumourigenesis and risk of cancer [44]. Notably, mutations in BRCA1 have been implicated in risk of hereditary cancers, such as breast, ovarian, pancreatic, and prostate cancer [45], whereas reduced levels of BRCA1 mRNA and protein have also been associated with sporadic tumours [46], [47], [48]. Additionally, other types of cancer have been ascribed to alterations in many DEGs detected in our analysis, such as *CDC6*, *BLM*, *RAD51*, and *MSH2* [39], [49], [50], [51]. These observations are in agreement with the proposed hypothesis of aetiological overlap between cancer and NSCL/P [15], [16], [17], [18].

We showed that transcriptional dysregulation of BRCA1 and its co-operators is associated with an accumulation of DSBs in NSCL/P cells, compared to controls. NSCL/P samples exhibited a heterogeneous behaviour, in which we observed three cellular phenotypes: individuals with increased DSB formation but efficient repair (subgroup I); those with increased DSB formation and deficient repair (subgroup II); and those with a similar pattern to the one observed among controls (subgroup III), which did not exhibit significant changes in the DSB profile. To better understand this variation, the mechanism by which H_2_O_2_ induces DSBs has to be taken into account. One possibility is that H_2_O_2_ generates the hydroxyl free radical OH°, a highly reactive oxygen species that afterwards induces formation of DNA single-strand breaks (SSBs) which, in turn, result in DSBs upon collapse of the replication fork during the S phase of the cell cycle. In this situation, cells accumulate one-ended DSBs, and repair likely occurs by homologous recombination [52], [53], [54], [55]. The detection of DEGs associated with processes presumed to prevent the oxidative generation of this type of DNA lesion and ensure its repair (i.e., oxidative stress, SSB repair, stabilisation of the replication fork, and DSB sensor signalling and repair via homologous recombination) explain the accumulation of DSBs observed in some of the NSCL/P samples. Therefore, we propose that the concurrent accumulation of G1/early S cells and elevated DSB signals at 24 hours, observed in NSCL/P subgroup II, reflects the inability of these cells to undergo homology-directed DSB repair. This is supported by the observed H_2_O_2_-dependent increment in apoptotic cells in this subgroup, and by the fact that NSCL/P cells that are able to repair the DSBs (subgroup I) did not display such accumulation in G1 and early S after 24 hours of treatment, being able to progress past early S and further into later stages of the cell cycle. Accordingly, controls and NSCL/P subgroup III did not show appreciable changes in DSB signal nor cell cycle distribution in the presence of H_2_O_2_, possibly because these cells do not possess alterations in anti-oxidative response and repair of DNA lesions. The finding that *CDC45L* fails to undergo up-regulation in NSCL/P cells exposed to H_2_O_2_ further suggests that deficiency in this type of DNA repair could play a role in the manifestation of the observed NSCL/P cellular phenotypes, as this DEG is involved in new DNA synthesis during homologous recombination [36] repair. H_2_O_2_ can also directly induce DSBs irrespective of cell cycle phase; consequently, deficiency of other repair pathways, such as non-homologous end-joining in G1 and G2 [56], would also be important. Yet, this does not seem to be the case, as the contribution of H_2_O_2_ in direct DSB formation is low [57]. Unexpectedly, in H_2_O_2_-treated cells we did not detect expression differences for the other DSB repair-related genes neither at 6 or 24 hours. This may have occurred because their transcriptional modulation could be required before the time points investigated in our experiments; for example, BRCA1 is known to act as an early detector and mediator in response to DSBs, and RAD51 is required for homology searching before DNA repair takes place [52], [58]. Nevertheless, we were able to confirm that NSCL/P cells display an impaired response to DNA damage, and the results suggest that oxidatively-generated DSBs may play an important role in this mechanism, providing a possible connection between this type of genotoxic insult and the aetiology of NSCL/P. Furthermore, the variation in response to H_2_O_2_ observed for the NSCL/P samples is in agreement with the genetic heterogeneity associated with the disease, indicating that these alterations are present in only some of the NSCL/P cases.

Previous research has reported a positive association between occurrence of NSCL/P and oxidative stress-initiating environmental factors [59]. Oxidative generation of DSBs and other types of DNA lesions have been reported to arise from many cleft-related environmental factors, such as maternal exposure to alcohol, nicotine, phenytoin, and valproic acid [59], [60], [61], [62], [63], [64], [65], [66], [67]. Among these, valproic acid has been reported to down-regulate homologous recombination DNA repair genes (e.g. *BRCA1*, *RAD51*, *BLM*) by decreasing E2F1 recruitment to its target promoters [68], which supports the hypothesis that this transcription factor could be at least in part responsible for dysregulating downstream genes through dysfunctional protein activity in the NSCL/P cells, as previously discussed. Moreover, folate deficiency has been related to orofacial clefts [69], and folate is essential for DNA biosynthesis, replication and repair [70], [71]; accordingly, it has been shown that genes related to DNA repair and cell cycle regulation, many of which were detected in our analysis, are differentially expressed in response to this molecule [72]. Importantly, if orofacial clefts are related to oxidative/genotoxic factors, they must act during embryonic development and affect the structures responsible for lip and palate formation, where abnormal cellular responses to these environmental insults are expected to play an important role in shaping susceptibility to NSCL/P.

If appropriate transcriptional regulation of the genes detected by our analyses is critical for normal lip and palate development, spatially and temporally co-ordinated expression of these genes would be expected in the embryonic precursors of these structures. Indeed, RNA *in situ* hybridisation assays using mouse embryos confirmed that *Brca1*, *Rad51* and *E2f1* are co-expressed within the mesenchyme of the facial primordia that contribute to the lip and the developing palatal shelves. *Brca1*, *Rad51* and *E2f1* were also detected in small domains within the ectoderm where they may act together with *Irf6* which is expressed throughout the facial epithelium [73], [74]. Therefore, *Brca1*, *Rad51* and *E2f1* are expressed during the critical stages of facial morphogenesis and dysregulation would be expected to impact on facial development. Their co-expression strongly supports the possibility that they function together as a molecular hub involved in facial growth and development. Another fact that supports this idea is that stem cells have increased DNA repair compared to differentiated cells [75]; thus, the decreased expression of these genes in NSCL/P cells may reflect problems during development. Furthermore, DNA repair and cell cycle-related genes are progressively down-regulated during orofacial morphogenesis, as suggested by the qRT-PCR experiments during palatogenesis here, and as reported in other works investigating the growth and fusion of facial prominences, and migrating neural crest cells [76], [77]. If these cellular systems are more active prior to differentiation of the craniofacial tissue, they must be important in a context of intense cellular proliferation or migration during the establishment, growth, and fusion of the embryonic facial structures, before differentiation takes place. Consequently, these embryonic structures must be more susceptible to the action of environmental DNA-damaging agents, and the effects would be expected to be exacerbated if dysregulation of the biological DNA repair processes revealed by the transcriptomic and functional assays here are taken into account. Therefore, the combined effects of transcriptional dysregulation and of environmental factors must be critical in a tissue-and time-specific manner, which can explain why orofacial clefts have not been observed in knockout animal models for pivotal DNA repair genes, including *E2F1*. Under these circumstances, we hypothesise that the inability to appropriately deal with DNA damage would result in disturbances in cell proliferation and/or lead to apoptosis, disrupting lip and palate morphogenesis.

In conclusion, we report here that gene networks governing cellular defences against DNA damage may play a role in the aetiology of NSCL/P, in accordance with the idea that orofacial clefts and cancer may have overlapping aetiologies. The identification of E2F1 as a putative regulator behind the expression profiles detected in this work reinforces the existence of one or a few upstream elements underlying dysregulation in NSCL/P cells. It is not yet possible to determine if the few NSCL/P-associated variants previously identified through GWAS [3], [4], [5], [6] can be accountable for dysregulating entire cellular functions as seen here; additionally, none of these variants are mapped to any of the DEGs identified in this work. Therefore, we speculate that alterations in a few unidentified upstream genetic or epigenetic regulators, combined with the effects of disease-associated variants, could be responsible for disturbances in regulatory or signalling events, such as those modulating activity of transcription factors like E2F1, or directly regulating entire pathways. Importantly, we do not presuppose that dysregulation of the biological processes described here is fully responsible for the pathogenesis of NSCL/P; instead, we believe that they are part of a variety of mechanisms, such as perturbations in extracellular matrix biology [8], [9], ultimately impairing orofacial morphogenesis. If regulatory anomalies are behind these disturbances, future research must focus on identifying such underlying genetic or epigenetic alterations that, upon interaction with environmental factors, result in cleft lip and palate. Consequently, a better understanding of the impact of these environmental agents, particularly those with genotoxic properties, will enable the development of preventive strategies in the future.

## Materials and Methods

### Ethics Statement

Ethical approval to extract stem cells from the dental pulp of deciduous teeth was obtained from the Biosciences Institute Research Ethics Committee (Protocol 037/2005) in the University of São Paulo. Samples were included only after signed informed consent by the parents or legal guardians. Those who declined to participate or otherwise did not participate were not disadvantaged in any way by not participating in the study. Care and use of mice were in compliance with the animal welfare guidelines approved by the Institute for Biosciences’ Animal Care and Use Committee and the King’s College Research Ethics Committee.

### Cell Cultures

Deciduous teeth were non-invasively obtained from children in exfoliation period. Specimens were kept in DMEM/High Glucose supplemented with 1% penicillin-streptomycin solution (Life Technologies), and taken to the laboratory to be processed. Control teeth were obtained from donors attending odontopaediatric clinics in São Paulo, Brazil, while NSCL/P teeth were obtained from patients enrolled for surgical treatment at SOBRAPAR Institute, Campinas, Brazil. We considered an individual to be affected by NSCL/P if no malformations other than clefting of the upper lip with or without cleft palate were present. RNA extracted from cell cultures derived from a total of 6 controls and 7 NSCL/P patients was used for microarray assays and quantitative real-time PCR. The same 7 NSCL/P patients and 2 novel control cell cultures in addition to 3 of the 6 aforementioned control samples were submitted to flow cytometry quantitation of γ-H2AX. Additional 4 NSCL/P and 6 control samples were used for qRT-PCR during exposure to H_2_O_2_ (See Table S5 for more details regarding the samples).

Dental pulp stem cell cultures were established according to previously published protocols. The primary culture establishment protocols used in our laboratory are reproducible and consistent with regard to the immunophenotype and differentiation potential of the cell populations [9], [78]. Cells were cultured in DMEM-F12 (Life Technologies) supplemented with 15% Foetal Bovine Serum (HyClone), 1% Non-essential aminoacids solution (Life Technologies), 1% penicillin-streptomycin solution (Life Technologies), in a humidified incubator at 37°C and 5% CO_2_. For storage, cells were frozen in medium containing 90% FBS and 10% DMSO (LGC Biotecnologia). For RNA extraction, frozen cells were thawed and grown until 80% confluent in a 75 cm^2^ culture flask. Microarray, qRT-PCR and flow cytometry experiments were conducted using cells between the 4^th^ and 8^th^ passage. During routine culture, cell populations exhibited spindle-shaped morphology and did not show significant morphological changes or cell death. We used asynchronous cells in all experiments.

In order to ensure that the transcriptional profiles were not biased by proliferative differences between controls and NSCL/P cells, 3 NSCL/P (F4243.1; F4244.1; F4293.1) and 3 control (F4217.1; F6119.1; F6032.1) samples were randomly chosen for proliferation assays. A total of 10^4^ cells/cm^2^ were seeded into 12-well plates (Corning). The following day, medium was changed and cells were harvested at days 0, 2, 3, 4, and 5 post-seeding, fixed in 1% paraformaldehyde, and counted using a flow cytometer (Guava). The results did not reveal significant differences between NSCL/P and controls (repeated measures two-way ANOVA, no interaction between factors; F = 0.3701, p>0.05; Fig. S2).

### RNA Extraction and Microarray Hybridisation

Total RNA isolation was performed with NucleoSpin RNA II kit (Macherey-Nagel), following manufacturer’s recommendations. RNA quality and concentration were assessed using Nanodrop 1000 and agarose gel electrophoresis. Only RNA samples with absorbance ratio 260/280>1.8, preserved rRNA ratio (28S/18S) and no signs of degradation were used.

Expression measurements were performed using the Affymetrix Human Gene 1.0 ST array, which interrogates 28,869 transcripts, followed by RNA labelling and hybridisation protocols as recommended by the manufacturer. After array scanning, quality control was performed with the GCOS software (Affymetrix) according to the manufacturer’s recommendations. Raw gene expression data are available at http://www.ncbi.nlm.nih.gov/geo/, under accession code GSE42589.

### Microarray Data Processing and Mining

Gene expression values were obtained using the three-step Robust Multi-array Average (RMA) pre-processing method, implemented in the Affy package in R/Bioconductor [79]. DEGs were acquired using two algorithms: SAM and Rank Products both included in the MeV (MultiExperiment Viewer) software. SAM is a t-test based method in which mean and variance are taken into account in DEG selection [80]. In contrast, Rank Products is a ranking-based method, which enables the identification of consistent differences, even if only in a subgroup of samples under analysis [81]. Genes selected by Rank Products do not necessarily exhibit homogeneous expression levels within test and control groups, and therefore, this analysis is suitable for detecting differential expression in complex diseases [9], [82]. Due to the complexity of the disease studied in this work, we decided to use both approaches in order to interrogate genes that are altered in all affected individuals analysed as well as genes altered in only a subgroup of them. Since SAM is a more conservative method, its p-value threshold was set at 0.05 while Rank Products’ was set at 0.01. Both were adjusted for multiple testing with the FDR (False Discovery Rate) method [83]. As the fold change calculation differs between the SAM and Rank Products methods, we calculated it for each gene by subtracting the average of the (log) control values from the average of the (log) case values (Avg(cases)-Avg(controls)).

### Transcriptome Analysis

We performed functional annotation and network analysis using IPA (http://www.ingenuity.com). We used the following parameters: Molecules per Network = 35; Networks per Analysis = 25; direct relationships only; “Ingenuity Expert Information” and “Ingenuity Supported Third Party Information” (including “miRNA-mRNA interactions”, “protein-protein interactions”, and “additional information”) data sources.

Supervised clustering was performed using EXPANDER (EXpression Analyzer and DisplayER - http://acgt.cs.tau.ac.il/expander/).We selected the probe matching the BRCA1 gene and set “expected cluster size” to 30. Gene Ontology (GO) enrichment analysis was executed with TANGO (Tool for ANalysis of GO enrichment), with the whole genome as the background set, and bootstrap-adjusted p-value = 0.001. Transcription factor binding site enrichment analysis was carried out using PRIMA (PRomoter Integration in Microarray Analysis), avoiding coding regions, with hit range between –1000 and +200, all genes as background, and Bonferroni-adjusted p-value threshold <0.05. These tools are also available in the EXPANDER software.

FANTOM4 (http://fantom.gsc.riken.jp/4/) was used to validate transcription factor-gene interactions. The FANTOM4 database contains transcriptomic and deep-CAGE information of differentiating THP-1 cell lines, as well as other published data, such as ChIP-chip [84]. We searched only for ChIP-chip experimental data, at t = 0 hours of differentiation.

### qRT-PCR Assays Performed on Cell Cultures

Two micrograms of total RNA extracted from each cell culture were converted into cDNA using Superscript II, according to the manufacturer’s recommendations. qRT-PCR reactions were performed in duplicates with final volume of 25 µL, using 20 ng cDNA, 2X SYBR Green PCR Master Mix, and 50 nM –200 nM of each primer. Fluorescence was detected using ABI Prism 7500 Sequence Detection System, under standard temperature protocol. Primer pairs were designed with Primer-BLAST (http://www.ncbi.nlm.nih.gov/tools/primer-blast/; primer sequences in Table S6), and their amplification efficiencies (E) was determined by serial cDNA dilutions expressed in log_10_ in which E = 10^−1/slope^. Expression of target genes was assessed relative to a calibrator cDNA (ΔCt). Finally, GeNorm v3.4 [85] was used to determine the most stable endogenous controls (among *GAPDH*, *HPRT1*, *SDHA*, *HMBS*), and calculate normalisation factors for each sample. The final expression values were determined based on a previous method [86], dividing E-^ΔCt^ by the corresponding normalisation factor. To compare the expression between control and NSCL/P groups, we applied a Student’s t-test with Welch’s correction. Primers and reagents were supplied by Life Technologies.

### DSB Quantitation and Assessment of Cell Cycle Distribution

NSCL/P and control cells were seeded into 6-well plates (Corning), in duplicates (10^4^ cells/cm^2^). The next day, cells were rinsed with PBS, and the culture medium was replaced by medium containing 100uM of freshly diluted H_2_O_2_ (Merck), followed by incubation for 6 and 24 hours in the dark, at 37°C/5% CO_2_, to stimulate DSB formation. Cells were harvested by trypsin incubation and fixed in 4% paraformaldehyde on ice for 15 minutes, followed by fixation in 70% ethanol overnight at −20°C. DSB quantification was performed based on a previously published protocol [87]. After complete fixation, cells were double-stained with PI (Propidium Iodide) and anti-γ-H2AX (Anti-phospho-H2A.X Ser139 clone JBW301 FITC conjugate, Millipore), in order to ascertain cell cycle distribution and DSB formation, respectively. Appropriate calibrators were applied for each individual (unstained sample; PI-stained- and anti-γ-H2AX -stained-only samples), and at least 5000 events were acquired. Data were analysed with Guava Express PRO software (Millipore) and gated to remove debris and cell clumps. To sort cells positively stained for γ-H2AX, we established a threshold using untreated cells of the same individual, below which ∼98% of the cells expressed γ-H2AX (intrinsic DSB formation); cells exposed to H_2_O_2_ and located above this threshold were considered positive for γ-H2AX. Sub-G1 events were quantified to estimate the number of apoptotic cells. H2AX profiles were compared using a Student’s t-test with Welch’s correction, whilst differences in sub-G1 cells were assessed using two-way ANOVA (subgroups×treatment) with Bonferroni post-tests for multiple comparisons.

### qRT-PCR and RNA in situ Hybridisation Studies in Mouse Embryos

Palatal shelves were dissected from CD1 mouse embryos at different stages of development: E11.5 (initial growth), E14.5 (period of fusion), and E17.5 (after complete formation), and divided into pools which were submitted to RNA extraction and conversion to cDNA. Gene expression levels were assessed by qRT-PCR, using appropriate mouse endogenous controls for normalisation (*B2m*, *Tbp*, *Tubb5* and *Ywhaz*; primer sequences in Table S6). Procedures were carried out as previously described. One-way ANOVA with Bonferroni post-tests was applied to compare mean expression values.

Whole-mount *in situ* hybridisation studies were carried out to E10.5, E11.5, E12.5 and E13.5 CD1 strain mouse embryos as previously described [88]. At E12.5 and E13.5 the palatal shelves were isolated to increase probe penetration. The embryonic tissues were treated with 10 µg/mL proteinase K for 20 (E10.5), 30 (E11.5), 45 (E12.5) and 60 minutes (E13.5). Whole-mounted embryos were fixed, embedded in 20% gelatin and were vibratome-sectioned at 40 µm. 900bp-1kb cDNA templates for ribroprobe synthesis were generated by PCR using the following primers: *Brca1* (5′GTCCTCGGCGCTTGGAAGTACG3′, 5′AACGACAGGCAGGTTCCCAGC3′), *Rad51* (5′GTGAGGATTTGGCGGGATTTCC3′, 5′CACTACTCAGGGCGGGGAGAGC3′); *E2f1* (5′CGCTGGTAGCAGTGGGCCAT3′, 5′ACCCCACGAGGCCCTTGACT3′) and were cloned in the pCRII-TOPO Vector. Antisense ribroprobe transcripts were synthesised with either T7 (*E2f1*) or Sp6 (*Bcra1* and *Rad51*) RNA polymerases.

## Supporting Information

Figure S1 **DNA repair genes are expressed in the developing palatal shelves.**Gene expression of key DNA repair genes was assessed in murine palatal shelves at various stages of development, using qRT-PCR. (*) p<0.05.
(TIF)

Figure S2 **NSCL/P and control cells exhibit similar proliferation profiles.** Proliferation assays were performed in 3 NSCL/P and 3 control cells, and revealed no significant differences (repeated measures two-way ANOVA, p>0.05).
(TIF)

Figure S3 **Negative control for the *in situ* hybridisation studies.** Sagittal views of negative sense controls performed on E11.5 and E12.5 mouse embryos, showing no staining.
(TIF)

Table S1 **List of DEGs obtained by comparing NSCL/P and control cells.** For DEGs selected by both SAM and Rank Products, the highest p-value between the two algorithms is shown. (*) Genes selected by SAM; (°) Genes selected by Rank Products. Information regarding probe sets lacking annotation are left blank.
(PDF)

Table S2 **Top enriched functions in the IPA interaction network.** Detailed list of the top functions enriched in the highest-scoring IPA network. The significance values for each biological function is a measure of the likelihood of that function being associated with the genes in the network due to chance, calculated using a right-tailed Fisher’s Exact Test.
(PDF)

Table S3 **Validation of the microarray assays.** Genes submitted to qRT-PCR to validate the microarray results, and their respective p-values. (*) Genes pertaining to the *BRCA1* similarity cluster.
(PDF)

Table S4 **DEGs involved in the oxidative generation and repair of DSBs.** Gene symbol, summarised function and literature reference of DEGs directly or indirectly involved in oxidative stress and homologous recombination repair of oxidatively-generated DSBs.
(PDF)

Table S5 **Cell cultures used in the study.** Laboratory code, gender, and clinical status of the samples used in the microarray assays and their validation by qRT-PCR, flow cytometry, and qRT-PCR during exposure to H_2_O_2_. (*) CL = Cleft Lip; CLP = Cleft Lip and Palate; UL = Unilateral Left; UR = Unilateral Right.
(PDF)

Table S6 **Primer sequences used for qRT-PCR experiments.**
(PDF)
