# Supplementary figures and images for: Susceptibility to DNA Damage as a Molecular Mechanism for Non-Syndromic Cleft Lip and Palate
Source: PLoS One. 2013 Jun 12;8(6):e65677. doi: 10.1371/journal.pone.0065677 (PMC3680497; doi:10.1371/journal.pone.0065677)

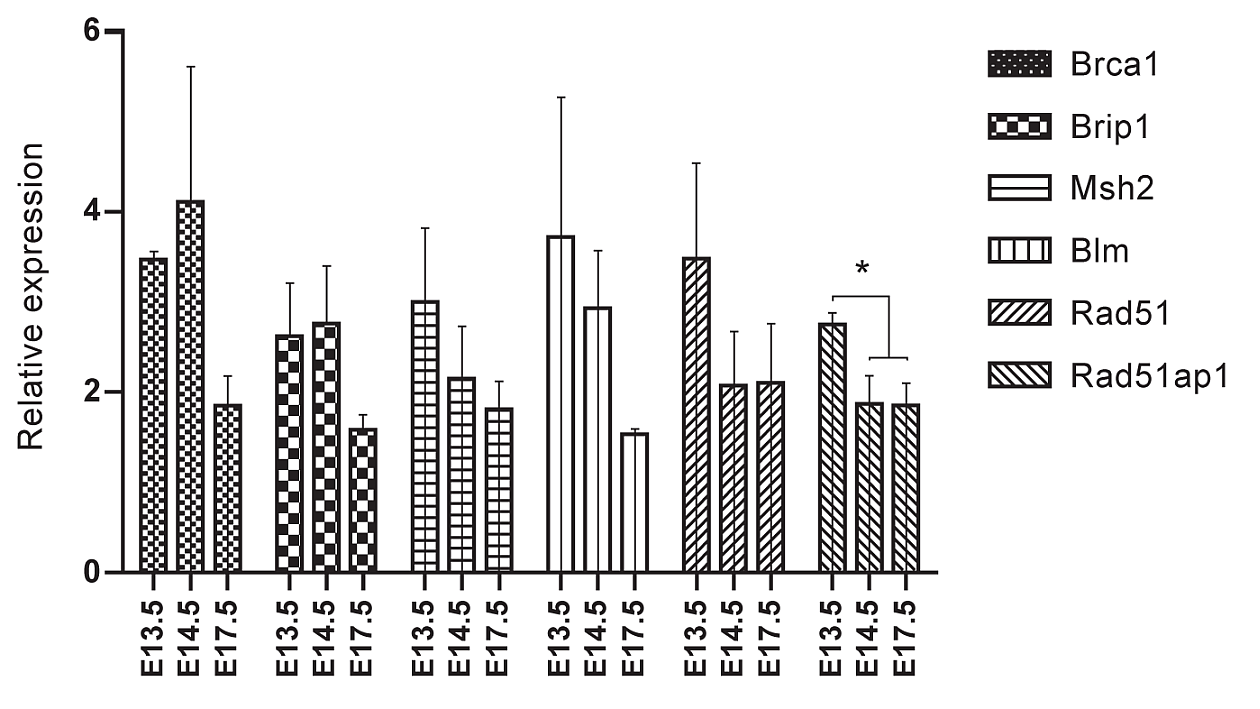

Supplement: Figure S1 — DNA repair genes are expressed in the developing palatal shelves.Gene expression of key DNA repair genes was assessed in murine palatal shelves at various stages of development, using qRT-PCR. (*) p<0.05. (TIF) [file pone.0065677.s001.tif]

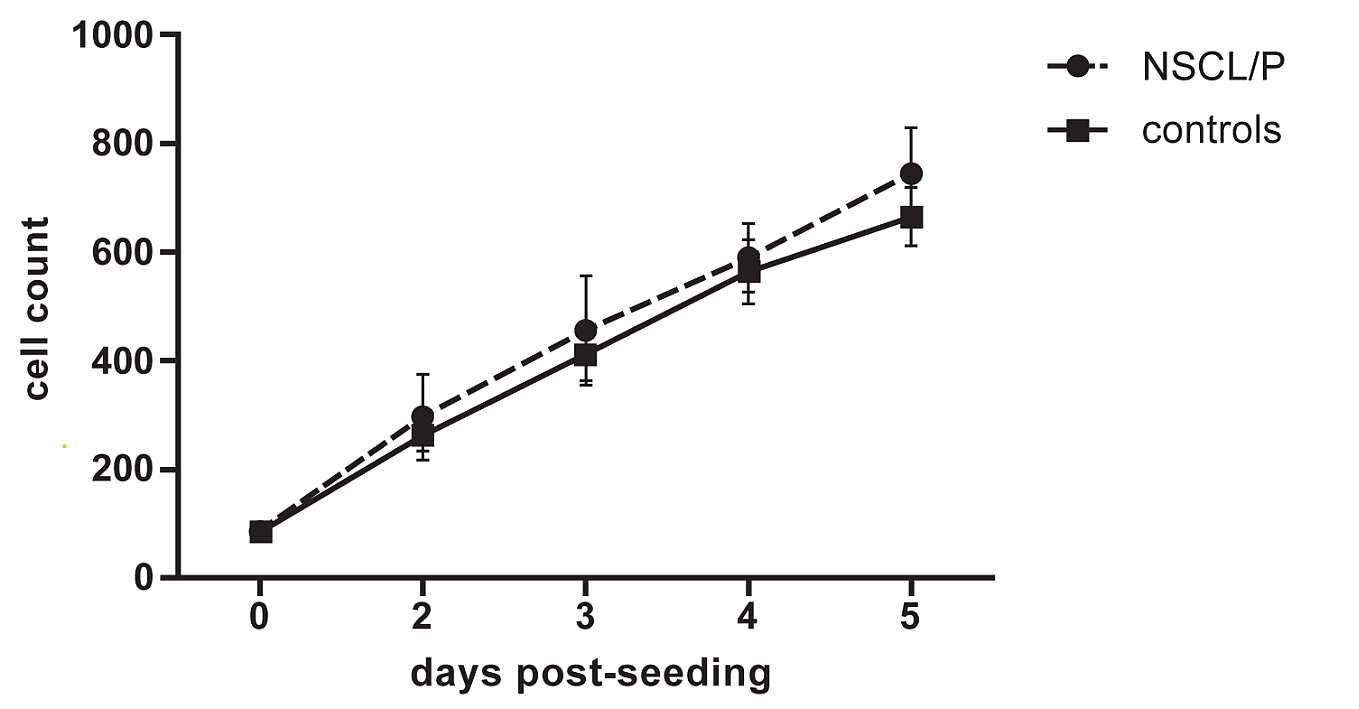

Supplement: Figure S2 — NSCL/P and control cells exhibit similar proliferation profiles. Proliferation assays were performed in 3 NSCL/P and 3 control cells, and revealed no significant differences (repeated measures two-way ANOVA, p>0.05). (TIF) [file pone.0065677.s002.tif]

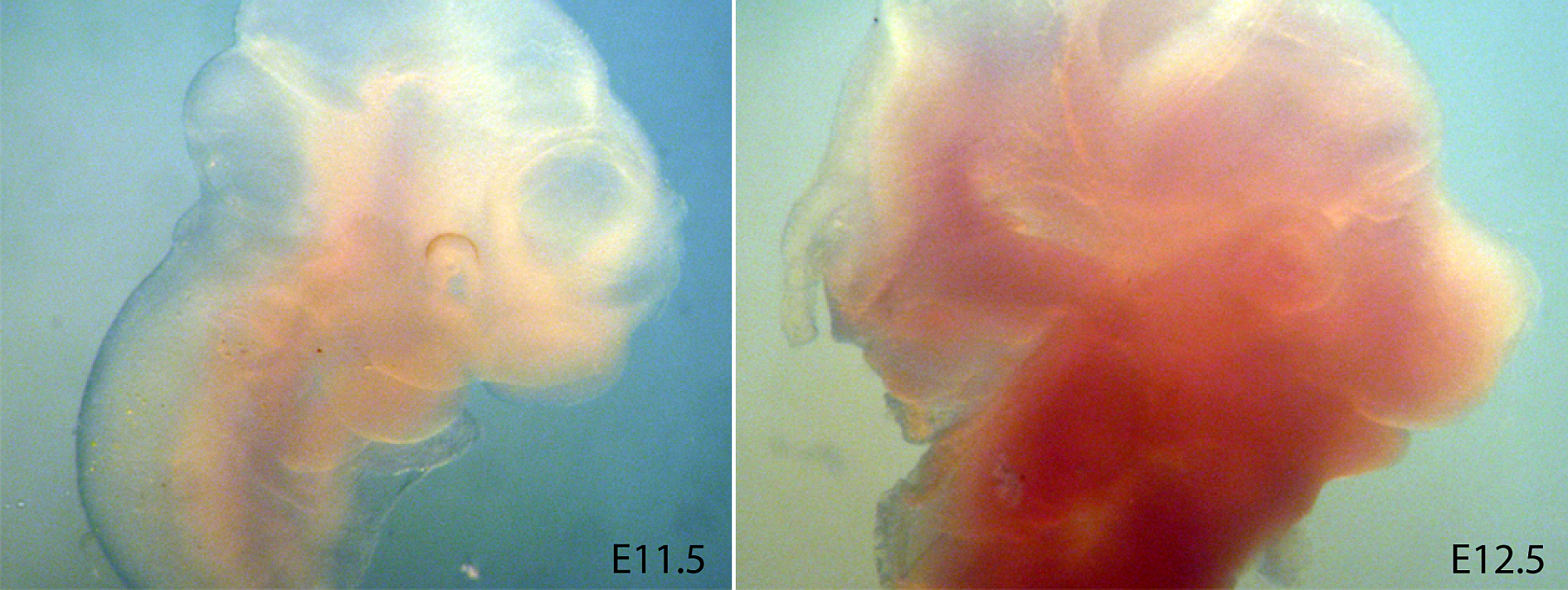

Supplement: Figure S3 — Negative control for the in situ hybridisation studies. Sagittal views of negative sense controls performed on E11.5 and E12.5 mouse embryos, showing no staining. (TIF) [file pone.0065677.s003.tif]
