# Supplementary material for: Susceptibility to DNA Damage as a Molecular Mechanism for Non-Syndromic Cleft Lip and Palate
Source: PLoS One. 2013 Jun 12;8(6):e65677. doi: 10.1371/journal.pone.0065677 (PMC3680497; doi:10.1371/journal.pone.0065677)
Supplement: Table S1 — List of DEGs obtained by comparing NSCL/P and control cells. For DEGs selected by both SAM and Rank Products, the highest p-value between the two algorithms is shown. (*) Genes selected by SAM; (°) Genes selected by Rank Products. Information regarding probe sets lacking annotation are left blank. (PDF) [file pone.0065677.s004.pdf]

Table SI: List of DEGs obtained by comparing NSCL/P and control cells

AffyID		Symbol	Entrez Gene Name	Fold change		P-value
8112045	° *	ESM1	endothelial cell-specific molecule 1	-5.330	±2.83	0.000
7922976	° *	PTGS2	prostaglandin-endoperoxide synthase 2 (prostaglandin G/H synthase and cyclooxygenase)	-3.709	±2.11	0.000
8067233	° *	PMEPA1	prostate transmembrane protein, androgen induced 1	-3.259	±2.01	0.000
8142981	° *	PODXL	podocalyxin-like	-2.920	±1.89	0.000
8116780	° *	DSP	desmoplakin	-2.736	±1.8	0.000
8100798	° *	SULT1B1	sulfotransferase family, cytosolic, 1B, member 1	-2.660	±2.37	0.000
7915612	° *	PTCH2	patched 2	-2.598	±1.51	0.000
8124527	° *	HIST1H1B	histone cluster 1, H1b	-2.587	±1.51	0.000
7973067	° *	PNP	purine nucleoside phosphorylase	-2.578	±1.45	0.000
8151871	° *	CCNE2	cyclin E2	-2.577	±1.44	0.000
8091243	° *	PCOLCE2 (includes EG:2657	procollagen C-endopeptidase enhancer 2	-2.568	±1.58	0.000
7924461	° *			-2.567	±1.54	0.000
7939237	° *	C11orf41	chromosome 11 open reading frame 41	-2.485	±1.39	0.000
8059376	° *	SERPINE2	serpin peptidase inhibitor, clade E (nexin, plasminogen activator inhibitor type 1), member 2	-2.463	±1.56	0.000
8105267	° *	ITGA2	integrin, alpha 2 (CD49B, alpha 2 subunit of VLA-2 receptor)	-2.440	±1.69	0.000
8045688	° *	TNFAIP6	tumor necrosis factor, alpha-induced protein 6	-2.436	±1.45	0.000
7952785	° *	OPCML	opioid binding protein/cell adhesion molecule-like	-2.431	±1.67	0.000
7948902	° *	SNHG1	small nucleolar RNA host gene 1 (non-protein coding)	-2.418	±1.49	0.000
7939215	° *	C11orf41	chromosome 11 open reading frame 41	-2.410	±1.34	0.000
8180255	° *			-2.404	±1.33	0.000
8180321	° *			-2.404	±1.33	0.000
8145793	° *	SNORD13	small nucleolar RNA, C/D box 13	-2.383	±1.45	0.000
7936968	° *	ADAM12	ADAM metallopeptidase domain 12	-2.339	±1.67	0.000
7999754	° *	XYLT1	xylosyltransferase I	-2.317	±1.43	0.000
7981084	° *	SERPINA9	serpin peptidase inhibitor, clade A (alpha-1 antiproteinase, antitrypsin), member 9	-2.307	±1.28	0.000
7974689	° *	DACT1	dapper, antagonist of beta-catenin, homolog 1 (Xenopus laevis)	-2.304	±1.47	0.000
8086752	° *			-2.279	±1.5	0.001
8124388	° *	HIST1H3A (includes others)	histone cluster 1, H3a	-2.273	±1.29	0.001
7970793	° *	SLC46A3	solute carrier family 46, member 3	-2.271	±1.47	0.000
7909568	° *	DTL	denticleless E3 ubiquitin protein ligase homolog (Drosophila)	-2.268	±1.35	0.000
7897801	° *	RNU5E-1	RNA, U5E small nuclear 1	-2.240	±1.32	0.001
7996260	° *			-2.233	±1.63	0.000
8007071	° *	CDC6 (includes EG:23834)	cell division cycle 6 homolog (S. cerevisiae)	-2.208	±1.31	0.001
7976812	° *	SNORD113-4	small nucleolar RNA, C/D box 113-4	-2.195	±1.61	0.001
8154233	° *	CD274	CD274 molecule	-2.184	±1.49	0.001
8156043	° *	PSAT1	phosphoserine aminotransferase 1	-2.151	±1.28	0.001
7940147	° *	FAM111B	family with sequence similarity 111, member B	-2.129	±1.24	0.001
7953218	° *	RAD51AP1	RAD51 associated protein 1	-2.120	±1.26	0.001
8126798	°	GPR116	G protein-coupled receptor 116	-2.119	±2.36	0.000
7919642	° *	HIST2H2AB	histone cluster 2, H2ab	-2.102	±1.2	0.002
8124391	° *	HIST1H2AB/HIST1H2AE	histone cluster 1, H2ae	-2.098	±1.24	0.002
8077499	° *	LINC00312	long intergenic non-protein coding RNA 312	-2.086	±1.51	0.001
7926259	° *	MCM10 (includes EG:307126	minichromosome maintenance complex component 10	-2.078	±1.2	0.002
8160431	° *	MIR31HG	MIR31 host gene (non-protein coding)	-2.060	±1.13	0.002
7950391	° *	PGM2L1	phosphoglucomutase 2-like 1	-2.060	±1.17	0.002
7943158	° *	SCARNA9	small Cajal body-specific RNA 9	-2.060	±1.27	0.002
7981978	° *	SNORD116-15	small nucleolar RNA, C/D box 116-15	-2.054	±1.23	0.002
8035838	° *	ZNF724P	zinc finger protein 724, pseudogene	-2.037	±1.15	0.002
8092640	° *	RFC4	replication factor C (activator 1) 4, 37kDa	-2.022	±1.08	0.002
8080847	° *	C3orf14	chromosome 3 open reading frame 14	-2.018	±1.11	0.002
8112376	° *	CENPK	centromere protein K	-2.018	±1.16	0.003
7981982	° *	SNRPN	small nuclear ribonucleoprotein polypeptide N	-2.004	±1.2	0.003
7981986	° *	SNRPN	small nuclear ribonucleoprotein polypeptide N	-2.004	±1.2	0.003
8083876	° *	SKIL	SKI-like oncogene	-2.002	±1.25	0.002
7927631	° *	DKK1	dickkopf 1 homolog (Xenopus laevis)	-2.002	±1.26	0.003
7922162	° *	SLC19A2	solute carrier family 19 (thiamine transporter), member 2	-2.001	±1.26	0.001
7998722	° *	SNORD60	small nucleolar RNA, C/D box 60	-1.997	±1.13	0.003
7906919	°	RGS4	regulator of G-protein signaling 4	-1.989	±1.76	0.002
7929438	° *	HELLS	helicase, lymphoid-specific	-1.976	±1.18	0.004
7981976	° *	SNORD116-14	small nucleolar RNA, C/D box 116-14	-1.967	±1.25	0.004
8071212	° *	CDC45	cell division cycle 45 homolog (S. cerevisiae)	-1.962	±1.08	0.004
7910997	° *	EXO1 (includes EG:26909)	exonuclease 1	-1.952	±1.14	0.004
8061471	° *	GINS1	GINS complex subunit 1 (Psf1 homolog)	-1.945	±1.12	0.004
8138527	° *	STEAP1B	STEAP family member 1B	-1.939	±1.2	0.004
8030978	° *	ZNF845	zinc finger protein 845	-1.938	±1.23	0.004
7919269	° *	GSTM2	glutathione S-transferase mu 2 (muscle)	-1.937	±1.09	0.004
7919349	° *	GSTM2	glutathione S-transferase mu 2 (muscle)	-1.937	±1.09	0.004
8124385	° *	HIST1H4A (includes others)	histone cluster 1, H4a	-1.937	±1.11	0.004
7930980	°	PPAPDC1A	phosphatidic acid phosphatase type 2 domain containing 1A	-1.934	±1.35	0.003
8140534	°	SEMA3C	sema domain, immunoglobulin domain (Ig), short basic domain, secreted, (semaphorin) 3C	-1.933	±1.41	0.003
8004144	° *	MIS12	MIS12, MIND kinetochore complex component, homolog (S. pombe)	-1.930	±1.05	0.004
7952339	° *	SNORD14C	small nucleolar RNA, C/D box 14C	-1.929	±1.21	0.005
8081241	° *	C3orf26	chromosome 3 open reading frame 26	-1.927	±1.13	0.004
7995354	° *	ORC6 (includes EG:23594)	origin recognition complex, subunit 6	-1.923	±1.08	0.004
8127989	° *	SNORD50B	small nucleolar RNA, C/D box 50B	-1.923	±1.1	0.005
7948904	° *	SNORD28	small nucleolar RNA, C/D box 28	-1.921	±1.28	0.005
7986068	° *	BLM	Bloom syndrome, RecQ helicase-like	-1.920	±1.08	0.004
7973896	° *	GSTM2	glutathione S-transferase mu 2 (muscle)	-1.918	±1.05	0.005
7978568	° *	GSTM2	glutathione S-transferase mu 2 (muscle)	-1.918	±1.05	0.005
7915926	° *	STIL	SCL/TAL1 interrupting locus	-1.915	±1.15	0.004
7908072	°	LAMC2	laminin, gamma 2	-1.914	±1.34	0.004
8141150	° *	ASNS	asparagine synthetase (glutamine-hydrolyzing)	-1.913	±1.11	0.004
7979710	° *	PLEK2	pleckstrin 2	-1.913	±1.12	0.004
7964271	° *	PRIM1	primase, DNA, polypeptide 1 (49kDa)	-1.909	±1.11	0.004
8015268	° *	KRT34	keratin 34	-1.900	±1.1	0.006
8095574	° *	DCK	deoxycytidine kinase	-1.899	±1.07	0.005
7964733	° *	RPSAP52	ribosomal protein SA pseudogene 52	-1.899	±1.22	0.005
8025402	° *	ANGPTL4	angiopoietin-like 4	-1.898	±1.06	0.004
8022674	° *	CDH2	cadherin 2, type 1, N-cadherin (neuronal)	-1.895	±1.26	0.003
7990345	°	SEMA7A	semaphorin 7A, GPI membrane anchor (John Milton Hagen blood group)	-1.883	±1.37	0.002

8142975 °	mir-29	microRNA 29a	-1.883	±1.31	0.005
7981990 °			-1.881	±1.27	0.005
8102787 ° *			-1.878	±1.05	0.006
8041867 ° *	MSH2	mutS homolog 2, colon cancer, nonpolyposis type 1 (E. coli)	-1.872	±1.05	0.006
7965094 ° *	E2F7	E2F transcription factor 7	-1.868	±1.08	0.005
8052382 ° *	FANCL	Fanconi anemia, complementation group L	-1.860	±1.06	0.007
8079153 ° *	ABHD5	abhydrolase domain containing 5	-1.853	±1.02	0.007
7982792 ° *	RAD51	RAD51 homolog (S. cerevisiae)	-1.851	±1.03	0.007
7898375 ° *	GSTM2	glutathione S-transferase mu 2 (muscle)	-1.851	±1.03	0.007
7898411 ° *	GSTM2	glutathione S-transferase mu 2 (muscle)	-1.851	±1.03	0.007
7912800 ° *	GSTM2	glutathione S-transferase mu 2 (muscle)	-1.851	±1.03	0.007
7912850 ° *	GSTM2	glutathione S-transferase mu 2 (muscle)	-1.851	±1.03	0.007
7919576 ° *	GSTM2	glutathione S-transferase mu 2 (muscle)	-1.851	±1.03	0.007
7974882 ° *	SYT16	synaptotagmin XVI	-1.839	±1.13	0.005
8144036 ° *	XRCC2	X-ray repair complementing defective repair in Chinese hamster cells 2	-1.832	±1.04	0.007
7976621 ° *	VRK1	vaccinia related kinase 1	-1.831	±1	0.007
8139632 ° *	FIGNL1	fidgetin-like 1	-1.824	±1.06	0.008
8097449 °	PCDH10	protocadherin 10	-1.823	±1.64	0.005
8003298 ° *	SLC7A5	solute carrier family 7 (amino acid transporter light chain, L system), member 5	-1.819	±1.09	0.007
8006187 ° *	ATAD5	ATPase family, AAA domain containing 5	-1.816	±1.11	0.007
7974337 *			-1.814	±1.14	0.000
8086880 ° *	CDC25A	cell division cycle 25 homolog A (S. pombe)	-1.807	±0.99	0.009
8015769 ° *	BRCA1	breast cancer 1, early onset	-1.802	±1.08	0.009
7983306 ° *	WDR76	WD repeat domain 76	-1.801	±1.09	0.009
8127987 °	SNORD50A	small nucleolar RNA, C/D box 50A	-1.801	±1.15	0.009
8117368 °	HIST1H4A (includes others)	histone cluster 1, H4a	-1.800	±1.13	0.007
8109830 ° *	CCDC99	coiled-coil domain containing 99	-1.798	±1.11	0.009
8132843 ° *	HAUS6	HAUS augmin-like complex, subunit 6	-1.797	±1.08	0.007
8055672 ° *	MMADHC	methylmalonic aciduria (cobalamin deficiency) cblD type, with homocystinuria	-1.794	±1.05	0.010
7934979 °	ANKRD1	ankyrin repeat domain 1 (cardiac muscle)	-1.790	±2.18	0.000
8114287 ° *	SPOCK1	sparc/osteonectin, cwcv and kazal-like domains proteoglycan (testican) 1	-1.784	±1.08	0.007
8152703 °	FBXO32	F-box protein 32	-1.784	±1.26	0.006
7917771 *	DNTTIP2	deoxynucleotidyltransferase, terminal, interacting protein 2	-1.782	±1	0.000
8017262 °	BRIP1	BRCA1 interacting protein C-terminal helicase 1	-1.779	±1.11	0.008
7914878 °	CLSPN	claspin	-1.773	±1.12	0.010
8152582 *	DSCC1	defective in sister chromatid cohesion 1 homolog (S. cerevisiae)	-1.772	±0.97	0.000
8053797 *	ANKRD36C	ankyrin repeat domain 36C	-1.768	±1.06	0.000
7922846 ° *	FAM129A	family with sequence similarity 129, member A	-1.763	±1.06	0.009
8124537 *	HIST1H3A (includes others)	histone cluster 1, H3a	-1.756	±1.05	0.000
7917976 *	SASS6	spindle assembly 6 homolog (C. elegans)	-1.754	±0.99	0.000
8115490 °	ADAM19	ADAM metallopeptidase domain 19	-1.751	±1.32	0.004
7997381 *	CENPN	centromere protein N	-1.748	±0.99	0.000
8067029 °	KCNG1 (includes EG:241794	potassium voltage-gated channel, subfamily G, member 1	-1.746	±1.28	0.005
7981953 *			-1.746	±0.99	0.000
7981966 *			-1.746	±0.99	0.000
8034512 *	SNORD41	small nucleolar RNA, C/D box 41	-1.737	±1.03	0.000
8151684 °	MMP16	matrix metallopeptidase 16 (membrane-inserted)	-1.721	±1.68	0.003
8054217 *	TXNDC9	thioredoxin domain containing 9	-1.719	±0.94	0.000
8001197 °	NETO2	neuropilin (NRP) and tolloid (TLL)-like 2	-1.715	±1.12	0.009
8042830 *	MTHFD2	methylenetetrahydrofolate dehydrogenase (NADP+ dependent) 2, methenyltetrahydrofolate cyclohydr	-1.711	±0.96	0.000
7905088 *	HIST2H2AC	histone cluster 2, H2ac	-1.709	±0.96	0.000
7970763 °	FLT1	fms-related tyrosine kinase 1 (vascular endothelial growth factor/vascular permeability factor receptor)	-1.708	±1.31	0.008
8066074 *	DSN1 (includes EG:1000029	DSN1, MIND kinetochore complex component, homolog (S. cerevisiae)	-1.701	±0.98	0.000
8042588 *	MPHOSPH10	M-phase phosphoprotein 10 (U3 small nucleolar ribonucleoprotein)	-1.700	±0.96	0.000
7962579 °	AMIGO2	adhesion molecule with Ig-like domain 2	-1.699	±1.19	0.008
7971077 °	POSTN	periostin, osteoblast specific factor	-1.691	±1.67	0.005
7968351 °	C13orf33	chromosome 13 open reading frame 33	-1.688	±1.4	0.006
7989915 *	TIPIN	TIMELESS interacting protein	-1.688	±0.95	0.000
7950933 °	NOX4	NADPH oxidase 4	-1.688	±1.48	0.007
7936322 *	GPAM	glycerol-3-phosphate acyltransferase, mitochondrial	-1.664	±0.93	0.000
7917182 °	ELTD1	EGF, latrophilin and seven transmembrane domain containing 1	-1.642	±1.35	0.007
8039484 °	IL11	interleukin 11	-1.622	±1.35	0.008
8145361 °	NEFM	neurofilament, medium polypeptide	-1.388	±2.23	0.003
7951271 °	MMP1 (includes EG:300339)	matrix metallopeptidase 1 (interstitial collagenase)	-1.228	±2.5	0.004
7985786 °	ACAN	aggrecan	-1.204	±2.3	0.006
8112971 °	HAPLN1	hyaluronan and proteoglycan link protein 1	-0.939	±2.16	0.009
7976567 °	BDKRB1	bradykinin receptor B1	1.129	±2.02	0.009
8045533 °			1.354	±1.46	0.007
8015179 °			1.384	±1.4	0.002
8019588 °			1.384	±1.4	0.002
7903214 °	LPPR4	lipid phosphate phosphatase-related protein type 4	1.391	±2.01	0.001
8176719 °	EIF1AY	eukaryotic translation initiation factor 1A, Y-linked	1.412	±2.09	0.002
8176375 °	RPS4Y1	ribosomal protein S4, Y-linked 1	1.431	±2.26	0.001
7999909 °	GPRC5B	G protein-coupled receptor, family C, group 5, member B	1.434	±1.38	0.009
7938225 °	OLFML1	olfactomedin-like 1	1.441	±1.04	0.009
7969202 °			1.464	±1	0.009
8176655 °	NLGN4Y	neuroligin 4, Y-linked	1.466	±1.7	0.003
8037240 °	PSG1	pregnancy specific beta-1-glycoprotein 1	1.480	±1.28	0.009
8013521 °			1.482	±1.08	0.009
8097288 °	FAT4	FAT tumor suppressor homolog 4 (Drosophila)	1.487	±1.07	0.009
7934185 °	C10orf54	chromosome 10 open reading frame 54	1.487	±0.87	0.008
7950005 °	MRGPRF	MAS-related GPR, member F	1.494	±1.14	0.007
8068361 °	SLC5A3	solute carrier family 5 (sodium/myo-inositol cotransporter), member 3	1.512	±0.97	0.009
8104758 °	NPR3 (includes EG:18162)	natriuretic peptide receptor C/guanylate cyclase C (atrionatriuretic peptide receptor C)	1.518	±1.16	0.005
7903358 °	VCAM1	vascular cell adhesion molecule 1	1.528	±1.56	0.009
8058664 °			1.536	±1.05	0.006
8037657 °	DMPK	dystrophia myotonica-protein kinase	1.563	±0.9	0.004
7896748 °			1.570	±1.89	0.001
7911335 °			1.571	±1.59	0.002
8165694 °			1.571	±1.59	0.002
8176578 °	USP9Y	ubiquitin specific peptidase 9, Y-linked	1.578	±1.99	0.001
7975076 °	HSPA2	heat shock 70kDa protein 2	1.588	±1.23	0.005
8150962 °	TOX	thymocyte selection-associated high mobility group box	1.635	±1.39	0.002
7932254 °	ITGA8	integrin, alpha 8	1.637	±1.06	0.004

8045835 °	GALNT5	UDP-N-acetyl-alpha-D-galactosamine:polypeptide N-acetylgalactosaminyltransferase 5 (GalNAc-T5)	1.639	±1.38	0.002
8176624 °	DDX3Y	DEAD (Asp-Glu-Ala-Asp) box polypeptide 3, Y-linked	1.644	±2.36	0.000
7926875 °	BAMBI	BMP and activin membrane-bound inhibitor homolog (Xenopus laevis)	1.651	±1.02	0.003
8141140 °	DLX5	distal-less homeobox 5	1.660	±1.25	0.003
8013523 °			1.662	±1.43	0.002
7962058 °	TMTC1	transmembrane and tetratricopeptide repeat containing 1	1.665	±1.66	0.002
7896750 °			1.669	±1.55	0.002
7984364 °	SMAD3	SMAD family member 3	1.689	±0.93	0.002
8156358 °			1.691	±1.9	0.001
8171297 °	MID1 (includes EG:10033095	midline 1 (Opitz/BBB syndrome)	1.691	±1.2	0.003
8113120 °			1.701	±1.22	0.002
8165707 °			1.701	±1.22	0.002
8129666 °	SLC2A12	solute carrier family 2 (facilitated glucose transporter), member 12	1.703	±1.04	0.002
8112668 °	GCNT4	glucosaminyl (N-acetyl) transferase 4, core 2	1.708	±1	0.002
7950810 °	SYTL2	synaptotagmin-like 2	1.718	±0.99	0.002
8095110 °	KIT	v-kit Hardy-Zuckerman 4 feline sarcoma viral oncogene homolog	1.722	±1.22	0.002
7911343 °			1.727	±1.38	0.002
8165703 °			1.727	±1.38	0.002
7970565 °			1.731	±1.06	0.002
8008965 °			1.738	±1.13	0.002
7962559 °	SLC38A4	solute carrier family 38, member 4	1.738	±1.23	0.002
8052355 °	EFEMP1	EGF containing fibulin-like extracellular matrix protein 1	1.759	±2.09	0.001
8013987 °			1.824	±1.21	0.001
8160346 °	PTPLAD2	protein tyrosine phosphatase-like A domain containing 2	1.831	±1.2	0.002
7899615 °	SERINC2	serine incorporator 2	1.843	±1.13	0.001
8165663 °			1.863	±1.74	0.001
7911337 °			1.865	±1.92	0.000
7973871 °			1.865	±1.92	0.000
8165696 °			1.865	±1.92	0.000
8102831 °	MGARP	mitochondria-localized glutamic acid-rich protein	1.874	±1.21	0.001
8165709 °			1.914	±1.26	0.001
7911339 °			1.952	±2.12	0.000
8165698 °			1.952	±2.12	0.000
8045804 °			2.012	±1.98	0.000
8102532 °	PDE5A	phosphodiesterase 5A, cGMP-specific	2.024	±1.4	0.001
8104746 °	NPR3 (includes EG:18162)	natriuretic peptide receptor C/guanylate cyclase C (atrionatriuretic peptide receptor C)	2.032	±1.38	0.000
7985317 °	KIAA1199	KIAA1199	2.088	±1.9	0.000
8057677 °	SLC40A1	solute carrier family 40 (iron-regulated transporter), member 1	2.169	±1.51	0.000
8100310 °			2.221	±1.88	0.000
7933204 °	C10orf10	chromosome 10 open reading frame 10	2.301	±1.43	0.000
8051583 °	CYP1B1	cytochrome P450, family 1, subfamily B, polypeptide 1	2.318	±1.84	0.000
8083887 °	CLDN11	claudin 11	2.430	±1.6	0.000
7912537 °	DHRS3	dehydrogenase/reductase (SDR family) member 3	2.441	±1.43	0.000
8006433 °	CCL2	chemokine (C-C motif) ligand 2	2.913	±2.11	0.000

(*) Genes selected by SAM; (°) Genes selected by Rank Products
