# Supplementary material for: Susceptibility to DNA Damage as a Molecular Mechanism for Non-Syndromic Cleft Lip and Palate
Source: PLoS One. 2013 Jun 12;8(6):e65677. doi: 10.1371/journal.pone.0065677 (PMC3680497; doi:10.1371/journal.pone.0065677)
Supplement: Table S2 — Top enriched functions in the IPA interaction network. Detailed list of the top functions enriched in the highest-scoring IPA network. The significance values for each biological function is a measure of the likelihood of that function being associated with the genes in the network due to chance, calculated using a right-tailed Fisher’s Exact Test. (PDF) [file pone.0065677.s005.pdf]

Table SII: Top enriched functions in the IPA interaction network

Function	Function Annotation	p-value	# Molec.
**Cell Cycle**			
recombination	DNA recombination	5.02E-09	7
recombination	recombination of cells	5.66E-05	3
homologous recombination	homologous recombination of DNA	4.42E-07	4
homologous recombination	homologous recombination of plasmid DNA	6.58E-05	2
homologous recombination	homologous recombination of cells	3.96E-02	1
checkpoint control	checkpoint control	4.96E-07	6
checkpoint control	checkpoint control of fibroblast cell lines	1.82E-04	2
DNA replication checkpoint	DNA replication checkpoint	1.99E-06	3
G2/M phase	G2/M phase	2.16E-05	6
G2/M phase	G2/M phase of tumor cell lines	6.16E-04	3
G2/M phase	G2/M phase of breast cancer cell lines	1.21E-03	2
G2/M phase	G2/M phase of cervical cancer cell lines	2.32E-02	1
G2/M phase	arrest in G2/M phase of breast cancer cell lines	3.32E-02	1
S phase checkpoint control	S phase checkpoint control of cervical cancer cell lines	1.08E-04	2
interphase	interphase	2.96E-04	9
interphase	arrest in interphase of fibroblast cell lines	1.79E-03	3
interphase	interphase of breast cancer cell lines	2.22E-03	3
interphase	arrest in interphase	1.19E-02	5
interphase	interphase of cervical cancer cell lines	1.29E-02	2
interphase	interphase of tumor cell lines	2.64E-02	4
interphase	arrest in interphase of tumor cell lines	4.69E-02	3
sister chromatid exchange	sister chromatid exchange	6.67E-04	2
sister chromatid exchange	sister chromatid exchange of DNA	3.70E-03	1
sister chromatid exchange	sister chromatid exchange of chromosomes	4.52E-03	1
sister chromatid exchange	sister chromatid exchange of cervical cancer cell lines	6.16E-03	1
G1/S phase transition	arrest in G1/S phase transition	9.41E-04	3
G1/S phase transition	arrest in G1/S phase transition of breast cancer cell lines	5.75E-03	1
meiosis	meiosis of germ cells	1.27E-03	3
meiosis	meiosis of primordial germ cells	2.47E-03	1
meiosis	meiosis of male germ cells	7.36E-03	2
morphology	morphology of spindle fibers	2.22E-03	2
S phase	S phase	2.33E-03	5
S phase	entry into S phase of tumor cell lines	8.63E-03	2
S phase	arrest in S phase of keratinocytes	9.43E-03	1
S phase	arrest in S phase	1.11E-02	2
S phase	entry into S phase	1.42E-02	3
S phase	S phase of fibroblast cell lines	1.55E-02	2
S phase	entry into S phase of cervical cancer cell lines	2.00E-02	1
S phase	re-entry into S phase of bone cancer cell lines	2.32E-02	1
S phase	entry into S phase of cancer cells	3.12E-02	1
S phase	arrest in S phase of fibroblast cell lines	3.72E-02	1
S phase	entry into S phase of breast cancer cell lines	4.71E-02	1
G1 phase	arrest in G1 phase of breast cancer cell lines	3.30E-03	2
G1 phase	arrest in G1 phase	1.13E-02	4
G1 phase	G1 phase of fibroblast cell lines	1.92E-02	2
G1 phase	arrest in G1 phase of carcinoma cell lines	4.79E-02	1
endomitosis	endomitosis of leukemia cell lines	3.70E-03	1
cell cycle progression	arrest in cell cycle progression of embryonic stem cell lines	4.11E-03	1
mitosis	delay in mitosis of breast cancer cell lines	4.11E-03	1
mitosis	entry into mitosis of embryonic cell lines	8.61E-03	1
mitosis	entry into mitosis of epithelial cell lines	8.61E-03	1
mitosis	entry into mitosis of kidney cell lines	1.15E-02	1
mitosis	entry into mitosis of colon cancer cell lines	1.51E-02	1
mitosis	mitosis of tumor cell lines	2.96E-02	2
mitosis	entry into mitosis of cervical cancer cell lines	3.00E-02	1

progression	progression of chromosomes	4.11E-03	1
DNA damage checkpoint	DNA damage checkpoint	5.26E-03	2
G2 phase	arrest in G2 phase of endometrial cancer cell lines	5.34E-03	1
G2 phase	arrest in G2 phase of embryonic stem cells	7.38E-03	1
G2 phase	arrest in G2 phase	1.26E-02	3
G2 phase	arrest in G2 phase of tumor cell lines	3.63E-02	2
G2 phase	arrest in G2 phase of lung cancer cell lines	4.19E-02	1
G2 phase	arrest in G2 phase of carcinoma cell lines	4.59E-02	1
organization	organization of chromosomes	6.19E-03	2
illegitimate recombination	illegitimate recombination of plasmid DNA	7.79E-03	1
senescence	senescence of fibroblasts	8.58E-03	2
aneuploidy	aneuploidy of fibroblasts	8.61E-03	1
aneuploidy	aneuploidy of cells	9.83E-03	2
abnormal morphology	abnormal morphology of meiotic spindles	9.43E-03	1
G2/M phase transition	arrest in G2/M phase transition	1.33E-02	2
reorganization	reorganization of chromatin	1.47E-02	1
homologous pairing	homologous pairing of DNA	1.76E-02	1
formation	formation of chromosomes	2.08E-02	1
segregation	segregation of chromosomes	2.34E-02	2
**DNA Replication, Recombination, and Repair**			
recombination	DNA recombination	5.02E-09	7
recombination	recombination of cells	5.66E-05	3
homologous recombination	homologous recombination of DNA	4.42E-07	4
homologous recombination	homologous recombination of plasmid DNA	6.58E-05	2
homologous recombination	homologous recombination of cells	3.96E-02	1
checkpoint control	checkpoint control	4.96E-07	6
checkpoint control	checkpoint control of fibroblast cell lines	1.82E-04	2
DNA replication checkpoint	DNA replication checkpoint	1.99E-06	3
repair	repair of DNA	7.53E-06	7
repair	repair of gene	1.31E-02	1
DNA damage response	DNA damage response of cells	8.46E-06	6
damage	damage of chromosomes	1.86E-05	4
metabolism	metabolism of DNA	4.47E-05	8
homologous recombination repair	homologous recombination repair of DNA	7.07E-05	3
formation	formation of chiasmata	8.55E-05	2
formation	formation of nuclear foci	8.94E-05	3
formation	formation of chromosome components	6.21E-04	3
formation	formation of sex bodies	4.11E-03	1
formation	formation of chromatin	5.34E-03	2
formation	formation of RAD51 nuclear focus	1.23E-02	1
formation	formation of chromosomes	2.08E-02	1
S phase checkpoint control	S phase checkpoint control of cervical cancer cell lines	1.08E-04	2
progression	progression of replication fork	1.14E-04	2
progression	progression of chromosomes	4.11E-03	1
replication	DNA replication	1.17E-04	6
replication	initiation of replication of DNA	2.51E-03	2
modification	modification of gene	2.22E-04	2
somatic hypermutation	somatic hypermutation	3.04E-04	2
breakage	breakage of chromosomes	3.91E-04	3
sister chromatid exchange	sister chromatid exchange	6.67E-04	2
sister chromatid exchange	sister chromatid exchange of DNA	3.70E-03	1
sister chromatid exchange	sister chromatid exchange of chromosomes	4.52E-03	1
sister chromatid exchange	sister chromatid exchange of cervical cancer cell lines	6.16E-03	1
breakdown	breakdown of chromosomes	6.97E-04	2
mutation	mutation of gene	6.97E-04	2
unwinding	unwinding of DNA	1.45E-03	2
unwinding	unwinding of DNA fragment	1.51E-02	1
mismatch repair	mismatch repair	1.71E-03	2
chromosomal instability	chromosomal instability	1.87E-03	2
double-stranded DNA break repair	double-stranded DNA break repair	2.36E-03	3

double-stranded DNA break repair	double-stranded DNA break repair of B-lymphocyte	3.70E-03	1
double-stranded DNA break repair	double-stranded DNA break repair of cells	7.41E-03	2
double-stranded DNA break repair	double-stranded DNA break repair of epithelial cells	9.02E-03	1
conversion	conversion of gene	2.88E-03	1
exchange	exchange of chromosomes	2.88E-03	1
exchange	exchange of sister chromatids	1.59E-02	1
joining	joining of DNA	4.09E-03	2
DNA damage checkpoint	DNA damage checkpoint	5.26E-03	2
organization	organization of chromosomes	6.19E-03	2
illegitimate recombination	illegitimate recombination of plasmid DNA	7.79E-03	1
quantity	quantity of nucleoprotein filaments	8.20E-03	1
abnormal morphology	abnormal morphology of meiotic spindles	9.43E-03	1
reorganization	reorganization of chromatin	1.47E-02	1
excision repair	excision repair	1.68E-02	2
homologous pairing	homologous pairing of DNA	1.76E-02	1
catabolism	catabolism of ATP	2.14E-02	2
segregation	segregation of chromosomes	2.34E-02	2
instability	instability of DNA	2.44E-02	1
ligation	ligation of DNA	3.24E-02	1
elongation	elongation of DNA	4.86E-02	1
**Cellular Compromise**			
damage	damage of chromosomes	1.86E-05	4
breakage	breakage of chromosomes	3.91E-04	3
breakdown	breakdown of chromosomes	6.97E-04	2
micronucleation	micronucleation of breast cancer cell lines	4.11E-03	1
atrophy	atrophy of motor neurons	8.20E-03	1
oxidative stress response	oxidative stress response of fibroblast cell lines	2.88E-02	1
