# Supplementary material for: Susceptibility to DNA Damage as a Molecular Mechanism for Non-Syndromic Cleft Lip and Palate
Source: PLoS One. 2013 Jun 12;8(6):e65677. doi: 10.1371/journal.pone.0065677 (PMC3680497; doi:10.1371/journal.pone.0065677)
Supplement: Table S3 — Validation of the microarray assays. Genes submitted to qRT-PCR to validate the microarray results, and their respective p-values. (*) Genes pertaining to the BRCA1 similarity cluster. (PDF) [file pone.0065677.s006.pdf]

Table SIII: Validation of the microarray assays

Gene	p-value
*CDC45L**	0.0058
PCOLCE2	0.0062
*BRCA1**	0.0083
*BRIP1**	0.0101
*DTL**	0.0113
*CDC25A**	0.0117
*RAD51AP1**	0.0118
E2F7	0.0124
*DKK1**	0.0166
CDH2	0.0169
LAMC2	0.0182
CCDC99	0.02
*BLM**	0.0209
*CDC6**	0.0238
DCK	0.0244
*RAD51**	0.0279
*HIST1H1B**	0.0496
PODXL	0.1062
MTHFD2	0.1113
ADAM12	0.1616
PCDH10	0.1679
HIST1H4B	0.2584
AMIGO2	0.3369
ITGA2	0.3459

(*) Genes pertaining to the BRCA1 similarity cluster
