# Supplementary material for: Susceptibility to DNA Damage as a Molecular Mechanism for Non-Syndromic Cleft Lip and Palate
Source: PLoS One. 2013 Jun 12;8(6):e65677. doi: 10.1371/journal.pone.0065677 (PMC3680497; doi:10.1371/journal.pone.0065677)
Supplement: Table S4 — DEGs involved in the oxidative generation and repair of DSBs. Gene symbol, summarised function and literature reference of DEGs directly or indirectly involved in oxidative stress and homologous recombination repair of oxidatively-generated DSBs. (PDF) [file pone.0065677.s007.pdf]

Table SIV: DEGs involved in the oxidative generation and repair of DSBs

Symbol	Function	Reference
GSTM2	ROS detoxification, glutathione metabolism	[26]
NOX4	Production of ROS	[27]
PTGS2	Peroxidase activity	[28]
SMAD3	oxidative stress response/TGFB-mediated growth inhibition	[29]
BRCA1	oxidative stress response/TGFB-mediated growth inhibition; Activation of base-excision repair/homologous recombination repair	[29], [31]
CLSPN	Stabilisation of the replication fork	[30]
TIPIN	Stabilisation of the replication fork	[30]
CDC45L	New DNA synthesis during DNA repair	[32]
MCM10	New DNA synthesis during DNA repair	[33]
GINS1	New DNA synthesis during DNA repair	[32]
