# Supplementary material for: Susceptibility to DNA Damage as a Molecular Mechanism for Non-Syndromic Cleft Lip and Palate
Source: PLoS One. 2013 Jun 12;8(6):e65677. doi: 10.1371/journal.pone.0065677 (PMC3680497; doi:10.1371/journal.pone.0065677)
Supplement: Table S5 — Cell cultures used in the study. Laboratory code, gender, and clinical status of the samples used in the microarray assays and their validation by qRT-PCR, flow cytometry, and qRT-PCR during exposure to H2O2. (*) CL = Cleft Lip; CLP = Cleft Lip and Palate; UL = Unilateral Left; UR = Unilateral Right. (PDF) [file pone.0065677.s008.pdf]

Table SV: Cell cultures used in the study

Code	Gender	Clinical Status*	Microarray	qRT-PCR validation	Flow cytometry	H2O2 qRT-PCR
F3334	female	control	X	X	X	X
F3347	male	control	X	X	X	X
F3960	female	control	X	X		
F4217	male	control	X	X	X	X
F4319	female	control	X	X		X
F4386	female	control	X	X		
F4243	male	CLP/UR	X	X	X	X
F4244	male	CLP/UR	X	X	X	X
F4245	male	CLP/UR	X	X	X	X
F4293	female	CL/UL	X	X	X	X
F4294	female	CL/UL	X	X	X	X
F4311	male	CL/UL	X	X	X	X
F4388	male	CLP/UR	X	X	X	X
F3333	male	control				X
F5541	male	control				X
F5703	male	control				X
F6032	female	control			X	X
F6119	male	control			X	X
F6681	male	control				X
F4281	male	CLP/UL				X
F4282	male	CLP/UL				X
F5640	female	CL/UL				X
F5720	male	CLP/UR				X

(*) CL = Cleft Lip; CLP = Cleft Lip and Palate; UL = Unilateral Left; UR = Unilateral Right
