# Supplementary material for: Susceptibility to DNA Damage as a Molecular Mechanism for Non-Syndromic Cleft Lip and Palate
Source: PLoS One. 2013 Jun 12;8(6):e65677. doi: 10.1371/journal.pone.0065677 (PMC3680497; doi:10.1371/journal.pone.0065677)
Supplement: Table S6 — Primer sequences used for qRT-PCR experiments. (PDF) [file pone.0065677.s009.pdf]

Table SVI: Primer sequences used for qRT-PCR experiments

Gene	Primer Forward (5'-3')	Primer Reverse (5'-3')
ADAM12	TCTTGCTGCCGGATTTGTGGTT	AAGGGCGCACACACCTTAGTTT
AMIGO2	AGGTGCAAGAGTGACAGACACGG	CTCTTTCCTGGGTTAGTCGGTGC
BLM	AGCACATTGTGTCAGTCAGTGGG	CTGTGGCCGTAAGAGCCATCAC
BRCA1	AGGTGAAGCAGCATCTGGGTGTG	TGCATGGTATCCCTCTGCTGAGTGG
BRIP1	AGTTCAGCTTCGGTTTGCTCGGG	AGCACACAGCTCGTAGGGGTTCA
CCDC99	GCCCTAGAGAAAGCTCGTGTAGC	CCACCTCTGCAAACAAAGAGTTGC
CDC25A	TTCCCTACCTCAGAAGCTGTTGGG	AGTGCAGGCAGCCACGAGATAC
CDC45L	GTGTGTGACACCCATAGGCCAG	TCTAACCGTGTGCGCTTCTCAG
CDC6	TCAGGAAGAGGTATCCAGGCCAG	AATACCAACACAATCATGGGGCCC
CDH2	CGCAGTGTACAGAATCAGTGGCGG	AGTCGATTGGTTTGACCACGGTG
DCK	TCAAGCCACTCCAGAGACATGCTT	TGCAGGAGCCAGCTTTCATGTT
DKK1	ATTGACAACTACCAGCCGTACCCG	AGTAATTCCCGGGGCAGCACATAG
DTL	TATGGAAGGTCTCCACACCCTGG	GTGAAGTCAGATGGACACCAGCAC
E2F7	CCTGTGCCAGAAGTTTCTAGCTCG	GCGTCTCCTTTCCACACCAAGAC
HIST1H1B	TCTTGCCACCATGTCGGAAACCG	CGGCAGCCTTCTTAGTTGCCTTC
HIST1H4B	ACCGAAAAGTGCTGCGGGATAAC	ACCGGAAATTCGCTTAACCCCAC
ITGA2	TGTTAGCGCYCAGTCAAGGCATTT	TGCTGCACTGCATAGCCAAACTGT
LAMC2	AGGTTGATACCAGAGCCAAGAACGCT	TCATCTACACTGAGAGGCTGGTCCAT
MTHFD2	TCTCTAATGTCTGCTTTGGCTGCC	CAACAGCTTCATTTCGAACTGCCG
PCDH10	CCAACGAGACTAAACACCAGCGAG	CTATGTCGGCTTCCTGGAATGCAG
PCOLCE2	TGCTGAACCAAACGAAAGAGGGGA	ATGTGCCACACACAAGTGACTCCT
PODXL	TTCCAGGAAGTCAGACCGTGGTC	ACTGACCCCTGCCTCCTTTAGTTC
RAD51	GGCAATGCAGATGCAGCTTGAAGT	TTATGCCACACTGCTCTAACCGTG
RAD51AP1	GCAGTGCCTTGTACAAAGATGGCT	GTGGTGACTGTTGGAAGTTCCTTCA
E2F1	CCGCCATCCAGGAAAAGGTGTG	TTCAGGTCGACGACACCGTCAG
GAPDH	TGCACCACCAACTGCTTAGC	GGCATGGACTGTGGTCATGA
HPRT1	TGACACTGGCAAAACAATGC	GGTCCTTTTCACCAGCAAGCT
SDHA	TGGGAACAAGAGGGCATCTG	CCACCACTGCATCAAATTCA
HMBS	GGCAATGCGGCTGCAA	GGGTACCCACGCGAATCAC
Brca1	AGAAGAAAGGGCCTTCACAATGTCC	ACCATTTGTAAGCTGCATTCCCGTG
Brip1	GGGACGCGGACACACAAGCAG	ACTTCCCGTGCTTGCCATCCAG
Msh2	TGGCATTTAAGGCTTCTCCCGGC	ACCCATAACGCCAACGGAAGCTG
Blm	AGTGCTGCAACGACCCCTCG	AGCCATGATCCTCATCTGGCATCC
Rad51	AGATGGAGCAGCCATGTTCGCTG	TCTCAGGTACAGCCTGGTGGTTG
Rad51ap1	AAGTCACCATCTCAAGCCAAGGC	TTTCTGAACCTTCACCAGTGGCG
B2m	TCGCGGTCGCTTCAGTCGTC	TTCTCCGGTGGGTGGCGTGA
Tbp	CCACACCAGCTTCTGAGAGC	GACTGCAGCAAATCGCTTGGG
Tubb5	GACAGTGTGGCAACCAGATCG	CTGCAGGTCGCTGTCACCGT
Ywhaz	TGAGCAGAAGACGGAAGGTGC	GCGAAGCATTGGGGATCAAGA
